# Phase I clinical study applying autologous immunological effector cells transfected with the interleukin-2 gene in patients with metastatic renal cancer, colorectal cancer and lymphoma

**DOI:** 10.1038/sj.bjc.6690800

**Published:** 1999-11

**Authors:** I G H Schmidt-Wolf, S Finke, B Trojaneck, A Denkena, P Lefterova, N Schwella, H-G Heuft, G Prange, M Korte, M Takeya, T Dorbic, A Neubauer, B Wittig, D Huhn

**Affiliations:** Department of Medicine, Division of Hematology/Oncology, Humboldt-Universität zu Berlin, Augustenburger Platz 1, Berlin 13353, Germany; Universitätsklinikum Carl Gustav Carus, Technische Universität Dresden, Dresden 01307, Germany; Centrum Somatische Gentherapie, Fachbereich Humanmedizin, Freie Universität Berlin, Berlin 14195, Germany

**Keywords:** interleukin-2, gene transfer, colorectal cancer, renal cancer, lymphoma, cytokine-induced killer cells

## Abstract

Natural killer-like T lymphocytes termed cytokine-induced killer (CIK) cells have been shown to eradicate established tumours in a severe combined immune deficient (SCID) mouse/human lymphoma model. Recently, we demonstrated that CIK cells transfected with cytokine genes possess an improved proliferation rate and a significantly higher cytotoxic activity as compared to non-transfected cells. Here, in a phase I clinical protocol, autologous CIK cells were generated from peripheral blood obtained by leukapheresis in patients with metastatic renal cell carcinoma, colorectal carcinoma and lymphoma. CIK cells were transfected with a plasmid containing the interleukin-2 (IL-2) gene via electroporation. Transfected cells generated IL-2 in the range of 330–1800 pg 10^−6^ cells 24 h^−1^ with a mean of 836 pg 10^−6^ cells 24 h^−1^. Ten patients received 1–5 intravenous infusions of IL-2-transfected CIK cells; five infusions with transfected CIK cells were given. In addition, the same patients received five infusions with untransfected CIK cells for control reasons. In three patients, WHO grade 2 fever was observed. Based on polymerase chain reaction of peripheral blood transfected cells could be detected for up to 2 weeks after infusion. There was a significant increase in serum levels of interferon gamma (IFN-γ), granulocyte–macrophage colony-stimulating factor (GM-CSF) and transforming growth factor beta (TGF-β) during treatment. Interestingly, there was also an increase in CD3+ lymphocytes in the blood of patients during therapy. In accordance, a partial increase in cytotoxic activity in peripheral blood lymphocytes (PBLs) was documented when patient samples before and after therapy were compared. Concerning clinical outcome, six patients remained in progressive disease, three patients showed no change by treatment, and one patient with lymphoma developed a complete response. In conclusion, we were able to demonstrate that CIK cells transfected with the IL-2 gene can be administered without major side-effects and are promising for future therapeutic trials. © 1999 Cancer Research Campaign


British Journal of Cancer (1999) **81**(6), 1009–1016
© 1999 Cancer Research Campaign
Article no. bjoc.1999.0800

### Phase I clinical study applying autologous immunological effector cells transfected with the interleukin-2 gene in patients with metastatic renal cancer, colorectal cancer and lymphoma

**IGH Schmidt-Wolf[1], S Finke[1], B Trojaneck[1], A Denkena[1], P Lefterova[1], N Schwella[1], H-G Heuft[1], G Prange[1], M Korte[1], M Takeya[3], T Dorbic[3], A Neubauer[2], B Wittig[3] and D Huhn[1]**

[1]Department of Medicine, Division of Hematology/Oncology, Humboldt-Universität zu Berlin, Augustenburger Platz 1, 13353 Berlin, Germany; [2]Universitätsklinikum Carl Gustav Carus, Technische Universität Dresden, 01307 Dresden, Germany; [3]Centrum Somatische Gentherapie, Fachbereich Humanmedizin, Freie Universität Berlin, 14195 Berlin, Germany

**Summary** Natural killer-like T lymphocytes termed cytokine-induced killer (CIK) cells have been shown to eradicate established tumours in a severe combined immune deficient (SCID) mouse/human lymphoma model. Recently, we demonstrated that CIK cells transfected with cytokine genes possess an improved proliferation rate and a significantly higher cytotoxic activity as compared to non-transfected cells. Here, in a phase I clinical protocol, autologous CIK cells were generated from peripheral blood obtained by leukapheresis in patients with metastatic renal cell carcinoma, colorectal carcinoma and lymphoma. CIK cells were transfected with a plasmid containing the interleukin-2 (IL-2) gene via electroporation. Transfected cells generated IL-2 in the range of 330–1800 pg $10^{-6}$ cells 24 $h^{-1}$ with a mean of 836 pg $10^{-6}$ cells 24 $h^{-1}$. Ten patients received 1–5 intravenous infusions of IL-2-transfected CIK cells; five infusions with transfected CIK cells were given. In addition, the same patients received five infusions with untransfected CIK cells for control reasons. In three patients, WHO grade 2 fever was observed. Based on polymerase chain reaction of peripheral blood transfected cells could be detected for up to 2 weeks after infusion. There was a significant increase in serum levels of interferon gamma (IFN-γ), granulocyte–macrophage colony-stimulating factor (GM-CSF) and transforming growth factor beta (TGF-β) during treatment. Interestingly, there was also an increase in CD3+ lymphocytes in the blood of patients during therapy. In accordance, a partial increase in cytotoxic activity in peripheral blood lymphocytes (PBLs) was documented when patient samples before and after therapy were compared. Concerning clinical outcome, six patients remained in progressive disease, three patients showed no change by treatment, and one patient with lymphoma developed a complete response. In conclusion, we were able to demonstrate that CIK cells transfected with the IL-2 gene can be administered without major side-effects and are promising for future therapeutic trials. © 1999 Cancer Research Campaign

**Keywords**: interleukin-2; gene transfer; colorectal cancer; renal cancer; lymphoma; cytokine-induced killer cells

Most patients with metastatic carcinomas have no hope for cure by standard forms of cancer therapy such as surgery, radiation and chemotherapy. A promising treatment strategy for many types of cancer is immunotherapy. The goal of this kind of therapy is to stimulate the immune system to recognize and kill cancer cells by modifying tumour cells or modifying the host response. Indeed, autologous immunological effector cells have been used successfully to treat patients with advanced stage malignancies (Rosenberg et al, 1986, 1990).

We reported a protocol generating large numbers of efficient cytotoxic effector cells termed CIK cells (Mehta et al, 1995; Schmidt-Wolf et al, 1991, 1994). Cytokine-induced killer (CIK) cells are non-major histocompatibility complex-restricted cytotoxic lymphocytes generated by incubation of peripheral blood lymphocytes with anti-CD3 monoclonal antibody, interleukin (IL)-2, IL-1 and interferon gamma (IFN-γ). CIK cells represent cells with high anti-tumour cytotoxicity in vitro and in vivo (Schmidt-Wolf et al, 1991). CIK cells possess enhanced cytotoxic activity as compared to standard lymphokine activated killer (LAK) cells (Lu et al, 1994; Margolin et al, 1997). The higher anti-tumour activity of CIK cells is mainly due to the higher proliferation rate of CD3 and CD56 double positive cells (Schmidt-Wolf et al, 1994). Because of the increase in cytotoxicity and high proliferative response, CIK cells have a 73-fold increase in total lytic units per culture as compared to IL-2-stimulated LAK cells. In a tumour colony assay these cells were capable of generating a log cell kill of 2.5–3.5 (Schmidt-Wolf et al, 1991). This represents an additional increase of about two logs of tumour cell kill as compared to LAK cells. As shown previously, the increase in cytotoxic activity has little toxic effect on a progenitor class of normal human bone marrow cells, since colony-forming unit granulocyte–macrophage (CFU-GM) activity of human bone marrow cells was only partially impaired (75% of control; Schmidt-Wolf et al, 1991). Furthermore, it has been shown that CIK cells are capable of eradicating an established tumour in a severe combined immune deficient (SCID) mouse human lymphoma model (Lu et al, 1994).

*Received 9 September 1998*
*Revised 23 April 1999*
*Accepted 30 April 1999*

*Correspondence to:* IGH Schmidt-Wolf

Present address: Med. Klinik und Poliklinik I, Rheinische Friedrich-Wilhelms-Universitat, Sigmund-Freud-Str. 25, D-53105 Bonn, Germany

LAK (Lotze et al, 1981; Welch et al, 1989) and CIK cells depend on exogenous addition of cytokines like IL-2, IL-7 or IL-12 for proliferation (Csipai et al, 1996; Zoll et al, 1998). The cytokines control the expansion of antigen-specific cells and influence the activity of cells within the immune system (Hickman et al, 1990). CIK lymphocytes transfected with a cytokine expression plasmid have shown improved cell proliferation and cytotoxic activity (Finke et al, 1998).

In this clinical phase I trial (Schmidt-Wolf et al, 1994) we generated autologous CIK cells, transfected CIK cells with the IL-2 gene and re-infused the cells into patients with metastatic colorectal carcinoma, renal cell carcinoma and lymphoma.

### MATERIALS AND METHODS

### Patient accrual

Details of the protocol have been described (Schmidt-Wolf et al, 1994). In brief, eligibility criteria consisted of patients with metastatic colon carcinoma, renal cancer, melanoma and lymphoma, age between 18 and 70 years, Karnofsky score of 70–100. Exclusion criteria included time interval from chemotherapy and cytokine treatment less than 28 days, creatinine higher than 265 μmol $l^{-1}$, bilirubin above 51 μmol $l^{-1}$, decompensated heart insufficiency, ventricular rhythm disorders, severe psychiatric disease, active hepatitis A, B, or C, HIV. Patients were treated as inpatients. Patients were informed of the investigational nature of this study and written consent in accordance with institutional policies was obtained before start of treatment. Approval of our local ethics committee was obtained.

### Generation of CIK cells

CIK cells were generated as described previously. In brief, non-adherent Ficoll separated human peripheral blood mononuclear cells were prepared and grown in RPMI-1640 medium (Gibco-BRL, Berlin, Germany), consisting of 10% fetal calf serum (FCS), FDA approved, (PAA, Cölbe, Germany), 25 mM HEPES, 100 U $ml^{-1}$ penicillin and 100 U $ml^{-1}$ streptomycin (Gibco-BRL, Germany). A total of 1000 U $ml^{-1}$ IFN-γ (Dr Rentschler, Laupheim, Germany) were added on day 0. After 24 h of incubation, 50 ng $ml^{-1}$ of an antibody against CD3 (Orthoclone OKT 3, Cilag GmbH, Sulzbach, Germany) and 100 U $ml^{-1}$ IL-2 (EuroCetus GmbH, Ratingen, Germany) were added. Cells were incubated at 37°C in a humidified atmosphere of 5% carbon dioxide and subcultured every 3rd day in fresh complete medium and IL-2 at $3 \times 10^6$ cells $ml^{-1}$.

### CELL LINES

For HLA-matched cytotoxicity assays various cell lines were used in this study. For patients with colorectal carcinoma we used HT 29 and CR 75 cells, for the patient with renal cell carcinoma we used A 704 cells, and for lymphoma patients we used LAM 53 cells. To monitor NK-activity of the patients we used K562 cells as targets in this assay. We received all cell lines from ATCC (Rockville, MD, USA), except CR 75, which was grown in our laboratory. The renal carcinoma cell line was grown in essential modified Eagle's medium (EMEM) with non-essential amino acids (Gibco-BRL), sodium pyruvate, 10% FCS (Gibco-BRL), 100 U $ml^{-1}$ penicillin and 100 U $ml^{-1}$ streptomycin. K562, HT 29, CR75 and LAM 53 cells were grown in RPMI-1640 (Gibco-BRL) with 10% FCS, 100 U $ml^{-1}$ penicillin and 100 U $ml^{-1}$ streptomycin.

### Expression plasmid for IL-2

The plasmid pCEP-IL-2 was constructed on the basis of the commercially available pCEP4 vector (Stratagene, NV Leek, The Netherlands). It contains a cytomegalovirus (CMV)-promoter-enhancer sequence, the cDNA from human IL-2 mRNA, a self-replication-origin (EBNA-1 and OriP), a gene for ampicillin- and for hygromycin-resistance and a SV-40 poly-adenosine (poly-A) signal sequence. The gene for episomal replication is under control of an Epstein–Barr virus (EBV) promoter and contains an EBV poly (A) signal sequence (Finke et al, 1998).

### Transfection of CIK cells

CIK cells were transfected via electroporation using the electroporation system easyject plus from Eurogentec, Seraing, Belgium. One $\times 10^7$ CIK-cells were suspended in 500 μl complete RPMI medium and were mixed with 30 μg pCEP-IL-2-plasmid. This mixture was transferred to a 4 mm electroporation cuvette and pulsed with a double pulse programme. Parameters for the first pulse were 650 V, 25 μF and 99 Ohm, for the second pulse 100 V, 1050 μF and 99 Ohm. After the pulse cells were transferred to complete RPMI medium at a density of $3 \times 10^6$ cells per ml, chloroquin at a final concentration of 100 μM was added to the cell suspension. Cells were incubated in a humidified atmosphere at 37°C overnight, then counted and dead cells were separated from the living cells by Ficoll separation before infusion.

Transfection efficiency was determined as described before (Finke et al, 1998). In brief, β-galactosidase assays were carried out 24 h after transfection of cells with an expression vector for bacterial β-galactosidase. The vector was constructed on the basis of the pCEP4 vector. Transfected cells were incubated with 5-bromo-4-chloro-3-indolyl-β-D-galactopyranoside (X-gal; Sigma, Deisenhofen, Germany) for 16–20 h at 37°C. Following incubation, aliquots were taken and the blue cells counted in a Neubauer chamber. Colourless cells were also counted and the percentage of transfected cells was calculated from the ratio between transfected and non-transfected cells.

### RNA extraction, reverse transcription and polymerase chain reaction

RNA was extracted from CIK cells and from PBLs using the RNeasy total RNA Kit (Qiagen, Hilden, Germany). RNA was reverse transcribed (RT) into cDNA using a standard procedure with random primers. No DNAase treatment was performed to remove residual plasmid DNA. Polymerase chain reaction (PCR) was performed using primers specific for IL-2 transcripts (Stratagene, Heidelberg, Germany). Twenty-five cycles of amplification were performed. Samples containing β-actin-specific primers (Stratagene) were used to monitor integrity and amount of cDNA – human β-actin-primers: TGACGGGGTC ACCCACACTG TGCCCATCTA, CTAGAAGCAT TTGCGGTGGA CGATGGAGGG; human IL-2 primers: GATTGTGATA TTGAAGGTAA AGATGGC, CTTC-CTTTAA CCTGGCCAGT GC.

Products of the PCR were analysed on a 3% agarose gel and photographed after ethidium bromide staining.

© 1999 Cancer Research Campaign

### Immunological studies

Most assays were performed using frozen cells. Therefore, cells taken at different time points could be tested in one assay. For flow cytometric analysis fresh cells were used.

*Immunofluorescence and flow cytometry studies*

Peripheral blood lymphocytes and CIK cells were stained using various monoclonal antibodies against human surface antigens. Antibodies used included antibodies against human CD3, CD4, CD8, CD16, CD19, CD25, CD28, CD56, LFA-1, ICAM-1 and HLA-DR (Immunotech Hamburg, Germany). Isotype-matched antibodies were used as controls. Stained cells were washed and subsequently analysed using a FACSCAN (Becton Dickinson). Background staining using irrelevant antibodies was less than 2%. A total of $10^4$ cells were analysed for each sample.

### Cytotoxicity assay

A Cyto Tox 96 non-radioactive cytotoxicity assay (Promega, Madison, WI, USA) was used to compare the cytotoxic activity of the transfected CIK cells with the non-transfected and to monitor the cytotoxic activity of the PBLs during the treatment. It was performed on days 1, 22, 25 and 43. This assay is a colourimetric alternative to the $^{51}Cr$ release assay. It quantitatively measures lactate dehydrogenase (LDH), which is released upon cell lysis in much the same way as $^{51}Cr$ is released. Released LDH in culture supernatants was measured in a 30-min incubation using a coupled enzymatic assay. The amount of colour formed is proportional to the number of lysed cells. Absorbance data were collected using a 96-well plate reader set to 490 nm. Five thousand target cells were plated in triplicate sets in a V-bottom 96-well tissue culture plate and incubated for 4 h with various ratios of effector to target cells. After incubation, 50 μl aliquots from all wells were transferred to a fresh 96-well plate. Fifty microlitres of the substrate mix was added to each well of the plate and incubated at room temperature for 30 min in the dark. Before measuring, 50 μl of a stop solution was added to each well. K562, CR 75, HT 29, A-704 and LAM 53 cells were used as targets. Every experiment was performed in triplicates and the mean value was calculated.

*Proliferation assay*

The proliferation assay ‘Easy for you’ (Biomedica, Vienna, Austria) was performed to compare the proliferation of transfected CIK cells with non-transfected cells and to determine the range of stimulatory effects of a mitogen (phytohaemaglutinine) on PBLs. Briefly, transfected and non-transfected cells were grown in 96-well plates for 7 days, fed once after 3 days, analysed using a chromophore substrate solution and read on a multiwell scanning spectrophotometer (enzyme-linked immunosorbent assay (ELISA) reader). Incubation with the substrate solution is 4 h, depending on the metabolic capacity of the cells. The yellow tetrazolium compound is converted to its red formazan derivative that has its maximum of absorbance at 490 nm. A medium control is subtracted in each well. Experiments were performed in triplicates and the mean value was calculated.

*ELISA*

IL-2 levels in conditioned medium were determined by an ELISA. The IL-2–ELISA kit was purchased from R&D Systems (Quantikine, Minneapolis, MN, USA). It detects soluble IL-2 down to 30 pg $ml^{-1}$ of IL-2. Briefly, microtitre plates were coated with a monoclonal antibody specific for IL-2 and incubated to bind IL-2 in cell culture supernatants. After several washes to remove unbound proteins, an enzyme-linked (horseradish peroxidase) polyclonal antibody was added that binds to the solid phase bound IL-2. After washing, the substrate solution was added and developing colour was measured using a multiwell plate reader set to 450 nm. The optical density of the samples was compared to a calibration curve. Standard samples according to WHO standards from the suppliers were used to generate calibration curves.

*Determination of cytokines other than IL-2*

In addition to IL-2 in the supernatant of transfected cells we determined the amount of IL-4, IL-12, granulocyte–macrophage colony-stimulating factor (GM-CSF), IFN-γ and tumour growth factor beta (TGF-β) in the serum of the patients on day 0, 4, 22, 25 and 43. All cytokine levels were determined by an ELISA and all kits were purchased from R&D systems, Quantikine. The assay procedures were analogous to the procedure described above.

### Delayed-type hypersensitivity

A commercially available recall-DTH test (Multi Test Merieux, Leimen, Germany) was administered before and after treatment. A positive skin-test reaction was defined as > 5 mm diameter induration after 48 h.

### Clinical outcome

A partial response was defined as a decrease of all measurable tumour manifestations of more than 50% for at least 4 weeks without new manifestations of disease. Stable disease was defined as no decrease of measurable tumour manifestations (WHO, 1979).

### Statistical analysis

Wilcoxon matched pairs test was used to analyse for statistical significance. A $P$-value $< 0.05$ was considered significant.

### RESULTS

### Clinical assessment of the course of disease and adverse effects

We monitored ten patients over a period of 43 days and performed various in vitro studies with PBLs isolated from patient’s blood collected on day 0, 4, 22, 25 and 43. In addition, we analysed patient’s serum for the presence of cytokines and tested the supernatant of transfected cells for the presence of IL-2.

### Patient characteristics

Ten patients were enrolled in our clinical protocol. Median age was 49.2 years (range 40–61). Nine patients were male, one female. Karnofsky score of all patients was 70 or above. Seven patients had metastatic colorectal carcinoma, two patients lymphoma, one patient renal cell carcinoma. Nine patients had metastases in lymph nodes, seven patients in the lung, five in the liver and two in other viscera.

© 1999 Cancer Research Campaign 

**Table 1** Patient characteristics, number of CIK cells administered and DTH reactivity

| Age | Sex | Diagnosis | HLA type administered | Total cells per cycle | Cells administered per cycle | Multitest- before therapy | Merieux after therapy | Fever |
|---|---|---|---|---|---|---|---|---|
| 61 | M | Colon | A2. A 25(10) | | $29.4 \times 10^8$ | | | |
| | | cancer | B 18, B50: | $51.9 \times 10^8$ | $22.5 \times 10^8$ | Negative | 11 mm/2 | + |
| | | | (21). Cw6, Bw6 | | | | | |
| 52 | M | Colon | A1. A24(9), B8, | | $11.4 \times 10^9$ | | | |
| | | cancer | B62(15), Cw3, | $12.5 \times 10^9$ | $10.6 \times 10^8$ | 5 mm/l | 6 mm/l | + |
| | | | Cw7. Bw6 | | | | | |
| 40 | M | Colon | A2. A24(9), B7 | $92.1 \times 10^8$ | $87.8 \times 10^8$ | | | |
| | | cancer | B27. Bw4, Bw6, Cw2, Cw7 | | $4.4 \times 10^8$ | Negative | Negative | – |
| 55 | M | Colon | A1. A9. B7, B8, | | $21.5 \times 10^8$ | | | |
| | | cancer | Bw6, Cw7 | $25.4 \times 10^8$ | $3.9 \times 10^8$ | 12 mm/l | 7 mm/3 | – |
| 46 | M | Colon | A2. A25(10), | | $5.5 \times 10^7$ | | | |
| | | cancer | B18. B51(5), | $21.9 \times 10^7$ | $16.4 \times 10^7$ | Negative | 2 mm/l | + |
| | | | Bw4. Bw6 | | | | | |
| 53 | M | Colon | A2. A28. B8(60). | | $8.4 \times 10^8$ | | | |
| | | cancer | B62(15). Bw4, | $14 \times 10^8$ | $5.6 \times 10^8$ | Negative | Negative | – |
| | | | Bw6. Cw3 | | | | | |
| 54 | F | Colon | A1. B7. B8 | | $2.7 \times 10^8$ | | | |
| | | cancer | Bw6. Cw7 | $6.8 \times 10^8$ | $4.1 \times 10^8$ | Negative | 2 mm/l | – |
| 41 | M | Follicular | A2.A30.(19).B13 | | $4.5 \times 10^7$ | | | |
| | | lymphoma | B62(15).Bw4.Bw6 | $4.8 \times 10^8$ | $4.3 \times 10^8$ | Negative | Negative | – |
| | | grade II | Cw4.Cw6.DR7. | | | | | |
| | | | DR12(5).DR52. DR53 | | | | | |
| | | | A1.A26(10).B27. | | $8.7 \times 10^8$ | | | |
| 54 | M | Renal | B57(17).Bw4. | $9.2 \times 10^8$ | $5.0 \times 10^7$ | 5 mm/l | ND | – |
| | | cancer | Cw1.Cw6 | | | | | |
| 54 | M | Follicular | A1.A33(19).B8. | | $4.5 \times 10^8$ | | | |
| | | lymphoma | B14.Bw6.Cw7.Cw8 | $18.4 \times 10^8$ | $13.9 \times 10^8$ | 9 mm/2 | 7 mm/2 | – |
| | | grade I | DR3.DR10.Dr52 | | | | | |
| | | | DR53 | | | | | |

One cycle of CIK cell infusions consisted of five infusions on consecutive days followed by a second cycle of five infusions after 3 weeks. DTH reactivity was performed on days 1 and 43 respectively. Results of Multitest Merieux are given as the sum of total diameter in mm/number of reactive agents before and after therapy. Fever was temperature WHO grade 2. M, male, F, female. ND, not done.

### Cells administered

Patients received one to five intravenous infusions of IL-2-transfected CIK cells and five infusions of untransfected CIK cells. The first cycle of infusions was given in 1 week from days 1 to 5, the second cycle was given from days 22 to 26. Patients were randomized into either receiving transfected or untransfected cells first.

Patients obtained a median of $35 \times 10^8$ CIK cells with a range of $2.2–125 \times 10^8$ (Table 1). Vitality was between 58.8 and 98.6% in the first three patients, and between 78.6 and 97.8% in the next seven patients. Vitality was determined by trypan blue stain.

### Clinical toxicity of treatment

Three patients developed WHO grade 2 fever that resolved the next day with or without the addition of antibiotics in all patients (Table 1). Sterility controls of CIK cells and blood cultures were negative at all times. No other adverse events were detected.

### Patient evaluation

Patients were evaluated before, during and after treatment. All parameters were scored for statistical significance. In summary, patients showed a statistically significant anemia on day 25 of therapy and on day 43 after therapy ($P = 0.012$ and 0.043 respectively), an increase in thrombocyte counts on days 22 and 43 ($P = 0.013$ and 0.041 respectively), an increase in potassium on days 4 and 22 ($P = 0.042$ and 0.028 respectively), an increase in alkaline phosphatase on day 43 ($P = 0.012$) and an increase in CRP on days 25 and 43 ($P = 0.028$ and 0.043 respectively) as compared to day 1 of therapy. There was no significant change in all other laboratory parameters including creatinine, bilirubin and tumour markers (data not shown).

### EBV status

Seven patients showed a positive EBV VCA-IgG and EBNA antigen before treatment. One of these patients showed an additional EBV VCA-IgM after treatment. One patient with negative EBV status before treatment showed a positive EBV VCA-IgG and EBNA antigen after therapy. One patient was not evaluable with respect to EBV (data not shown).

### DTH reactivity

Multitest Merieux was performed before and after treatment (Table 1). An increase in the reactivity against bacterial antigens was seen in three of ten patients.

### Patient outcome

All ten patients were in progressive disease when entering our protocol. With respect to our protocol, clinical outcome was based mainly on comparison of CT scans before and after treatment. Six

 *© 1999 Cancer Research Campaign*

patients remained in progressive disease, three patients showed no change by treatment. In one patient with follicular lymphoma grade I, a pre-existing bone marrow involvement as the only sign of disease resolved after CIK cell therapy. This was scored as a complete clinical response.

### In vitro immunological responsiveness

### *Determination of gene transcription using PCR*

We looked for the presence of IL-2 transcripts in the blood of all patients during treatment. Transcription of IL-2 cDNA was shown using RT-PCR. Further, we looked for the presence of IL-2 cDNA in transfected and non-transfected CIK cells (treatment cycles I and II) of all patients. Non-transfected CIK cells were shown to contain only small amounts of IL-2 mRNA in contrast to cells transfected with the IL-2 vector, which showed successful transfection in every case (data not shown). Based on PCR of peripheral blood transfected cells could be detected for up to 2 weeks after infusion. Concerning RNA of the PBL, one patient showed no IL-2 mRNA, one patient showed a band only on day 25 of the treatment and one patient showed a band on day 0, which disappeared during the time of observation. In seven patients, IL-2 mRNA was detectable on each evaluation day during treatment (data not shown).

### *Cytokine production of CIK cells*

Successfully transfected CIK cells produced a mean of 836 pg $10^{-6}$ cells 24 $h^{-1}$ IL-2. The range between patients was 330–1800 pg $10^{-6}$ cells 24 $h^{-1}$ as measured by ELISA (data not shown). The percentage of actually transfected CIK cells varied between 5 and 15%. However, it was difficult to transfect CIK cells derived from the two lymphoma patients. Here, we measured 75 and 0 pg $10^{-6}$ cells 24 $h^{-1}$ IL-2 respectively. In non-transfected cells, IL-2 expression was below 30 pg $ml^{-1}$. Also, when CIK cells were transfected with vector constructs of identical basis but containing a β-galactosidase gene, IL-2 production was below 30 pg.

### *Cytokine profile of the serum from patients*

In addition to IL-2, we assayed the serum of the patients for IL-4, IL-12, IFN-γ, GM-CSF and TGF-β. In no case was a production of IL-2, IL-4 or IL-12 detectable. None of the patients had detectable amounts of IFN-γ in serum on day 0, but in six patients IFN-γ was detectable during treatment. In patient 8 (P8) we found 67 pg $ml^{-1}$ on day 43, the other patients showed varying amounts of IFN-γ, in particular on days 22, 25 and 43. Highest amounts were in the range of 16–49 pg $ml^{-1}$. This increase in single patients was statistically significant, with a *P*-value of 0.0369 (Table 2).

**Table 2** Cytokine production in the serum of the patients on day 0 and day 43

| | pg $ml^{-1}$ on day 0 (mean value) | pg $ml^{-1}$ on day 43 (mean value) | *P*-value |
|---|---|---|---|
| IL-2 | 0 | 0 | |
| IL-4 | 0 | 0 | |
| IL-12 | 0 | 0 | |
| IFN-gamma | 0 | 13.7 | 0.0369 |
| GM-CSF | 8.1 | 28.7 | 0.0312 |
| TGF-β | 16.970 | 35.400 | 0.0096 |

In two patients we found 40 pg $ml^{-1}$ GM-CSF in the serum on day 0. In one patient, the amount of GM-CSF increased during treatment to a maximum value of 184 pg $ml^{-1}$ on day 43. In all patients GM-CSF increased from 8.1 to 28.7 during treatment, with a *P*-value of 0.0312 (Table 2). CIK cells expressed a mean of 700 pg $ml^{-1}$ $10^{-6}$ cells of GM-CSF.

We detected a high TGF-β level in the serum of all patients (> 20 ng $ml^{-1}$) with the exception of P4 (725 pg) and P5 (9 ng). However, there was a significant increase in the expression of TGF-β in all patients during treatment when we calculated day 0 versus day 25 or 43, with a *P*-value of 0.0171 and 0.0096 respectively (Table 2).

### *Expression of surface antigens on PBLs during treatment*

PBLs were stained with various monoclonal antibodies as outlined above. There was a significant increase of CD3+CD8+ PBLs in relative numbers from 20% to 25% (mean value) in all patients on day 25 with a *P*-value of 0.0049 (Figure 1); however, the increase in absolute numbers was not statistically significant. CD3+ and CD4+ cells increased from 507±182 and 237±162 to 708±340 and 327±204 per pl blood on day 22 ($P < 0.05$ for CD3) respectively (Table 3). In addition, there was an increase in CD25+ cells on day 25. Significance was not seen at any other time points.

### *Expression of surface antigens on CIK cells*

Concerning the expression of surface molecules on transfected and non-transfected CIK cells surface molecules remained unaltered. However, there was a significant decrease in the expression of CD3+CD56+ cells from 14 to 6% with a *P*-value of 0.0391, and of LFA-1+ cells from 96% to 65% with a *P*-value of 0.0167 (data not shown).

### *Proliferation of CIK cells*

When transfected CIK cells were compared with non-transfected cells (in vitro experiments), CIK cells transfected with the IL-2 gene showed an increased proliferation rate as compared to non-transfected cells (0.371±0.148 versus 0.161±0.043). This enhanced proliferation rate was statistically significant, but cells proliferated less as compared to positive controls fed with exogenous IL-2 (mean value 0.606±0.211). The *P*-value was 0.0415 when we calculated significance of CIK cells without transfection versus transfected cells. These results correlate well with results we obtained when transfecting CIK cells with IL-7 via receptor-mediated gene transfer (Finke et al, 1998).

### *Cytotoxicity of PBLs collected during treatment*

The cytotoxic effect of PBLs was monitored during the treatment using various HLA-matched carcinoma cell lines as targets in a non-radioactive cytotoxicity assay. Cytotoxic activity of PBLs increased during treatment. This was true either against HLA-matched carcinoma cell lines as well as against K562 cells. We used effector to target cell ratios of 2, 5, 10, 20:1 cells. In every case the cytotoxicity of the PBLs increased during the treatment as compared to their activity before treatment. However, only at single effector to target ratios was this increase statistically significant. At the 20:1 effector to target cell ratio we found a mean value of 22.5±26% cell killing for the PBL on day 0 and a mean value of 35±21% on day 43, with a *P*-value of 0.029 against K562.

*© 1999 Cancer Research Campaign*

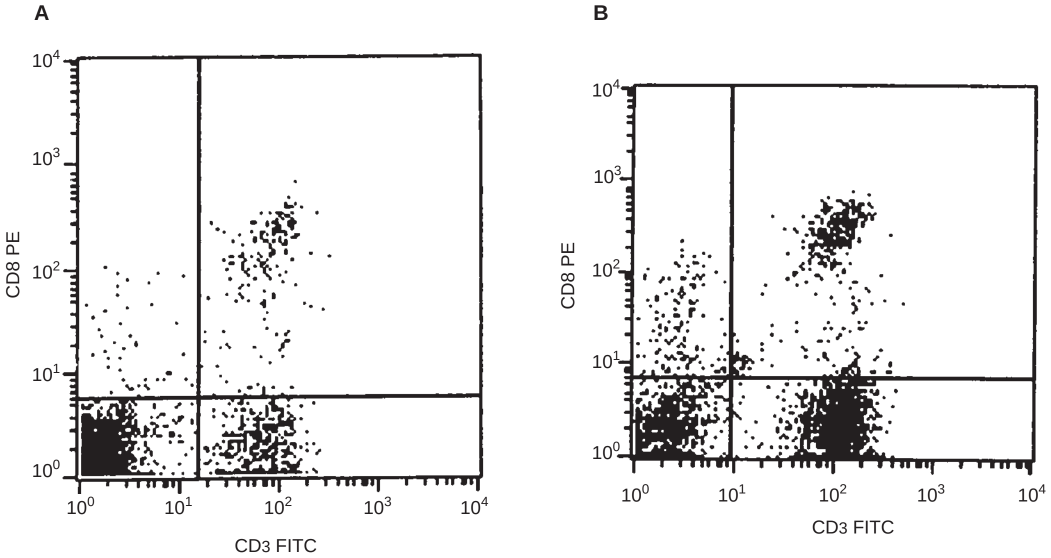

**Figure 1** Flow cytometry of PBL during treatment. PBLs were stained with various antibodies and analysed by flow cytometry as described in Material and Methods. For example, cell surface expression of CD3 and CD8 was determined on day 0 (**A**) and day 25 (**B**). Cells stained with an irrelevant antibody were used as negative control. Here, data from one representative patient are shown

**Table 3** Flow cytometry studies

| | PBL day 0 | PBL day 4 | PBL day 22 | PBL day 25 | PBL day 43 |
|---|---|---|---|---|---|
| CD4+CD3+ | 237±162 | 247±149 | 327±204 | 205±136 | 270±183 |
| CD4+ | 237±162 | 247±149 | 327±204 | 205±136 | 270±183 |
| CD3+ | 507±182 | 458±202 | 708±340 | 479±179 | 529±215 |
| CD3+CD8+ | 203±91 | 167±79 | 272±272 | 214±222 | 183±75 |
| CD3+ | 487±212 | 449±132 | 721±258 | 402±247 | 486±226 |
| CD8+ | 314±162 | 247±114 | 395±272 | 291±205 | 291±107 |
| CD3+CD19+ | 10±10 | 9±17 | 14±13 | 26±25 | 11±10 |
| CD3+ | 487±202 | 458±140 | 531±381 | 402±247 | 475±226 |
| CD19+ | 132±70 | 106±70 | 109±68 | 120±119 | 140±97 |
| CD3+CD56+ | 41±30 | 18±17 | 41±40 | 51±34 | 32±43 |
| CD3+ | 497±192 | 441±144 | 694±231 | 410±196 | 496±205 |
| CD56+ | 152±111 | 167±79 | 163±136 | 120±59 | 162±107 |
| CD16+CD56+ | 41±40 | 53±44 | 54±40 | 60±51 | 76±86 |
| CD16+ | 101±91 | 88±70 | 136±149 | 128±145 | 129±118 |
| CD56+ | 101±81 | 70±61 | 136±136 | 103±42 | 119±107 |
| HLA-DR+CD25+ | 20±20 | 18±17 | 27±27 | 51±42 | 22±21 |
| HLA-DR+ | 183±101 | 123±79 | 231±149 | 222±119 | 248±129 |
| CD25+ | 61±50 | 53±44 | 68±40 | 111±102 | 65±53 |
| LFA 1+ | 730±233 | 617±211 | 776±449 | 590±273 | 820±194 |
| ICAM 1+ | 649±354 | 484±317 | 544±36 | 410±333 | 518±420 |
| CD28+ | 446±192 | 344±167 | 544±285 | 316±196 | 356±237 |

PBLs were stained on day 0, 4, 22, 25 and 43 with various antibodies and analysed by flow cytometry. A total of $10^4$ cells were analysed for each sample and background staining was usually less than 2%. Data are presented as mean cell number per pl blood of ten separate experiments.

We found a statistically significant increase from 8.2±9.7% on day 0 to 13.2±9.6% on day 25, with a *P*-value of 0.016 at an effector to target ratio of 10:1 against HLA-matched tumour cells. Figure 2A shows the cytotoxic activity of the PBLs against K562, Figure 2B against HLA-matched tumour cells.

### *Cytotoxicity of CIK cells*

In CIK cells we found an increase in cytotoxic activity when comparing transfected to non-transfected cells. This was true when using HLA-matched tumour cells as targets and this increase was statistically significant for the effector to target ratio of 5:1. Cytotoxic activity was increased from 3.8% to 9.7% with a *P*-value of 0.0331. When using K562 cells as targets the cytotoxic activity remained unaltered (data not shown).

### DISCUSSION

The ability of malignant cells to survive exposure to cytotoxic agents is a major obstacle to cure in cancer patients. Immunological effector cells such as LAK cells (Grimm et al, 1982; Lynch et al, 1990), TILs (Rosenberg et al, 1986) or NK-like T-cells, termed cytokine-induced killer (CIK) cells (Mehta et al,

 © 1999 Cancer Research Campaign

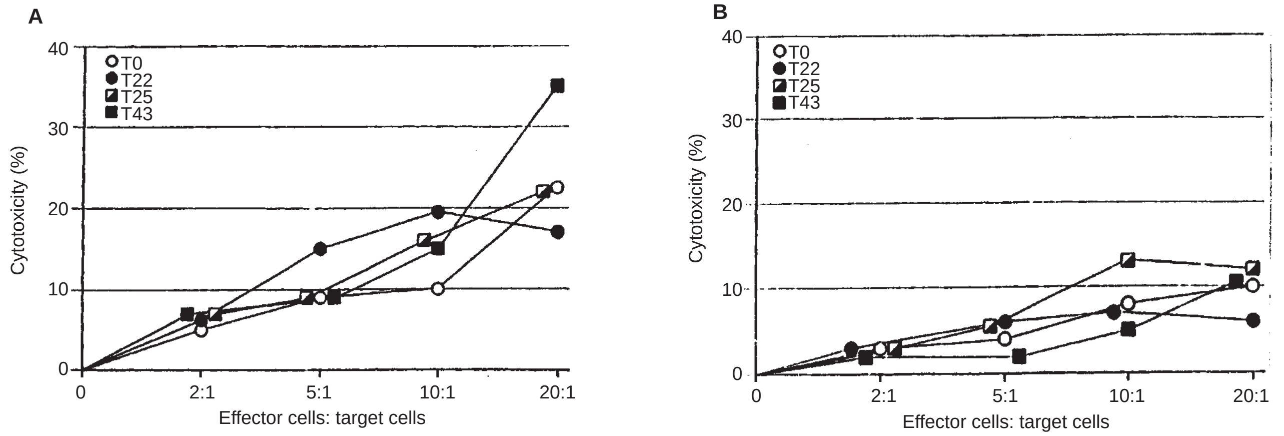

**Figure 2** Cytotoxicity of PBL during treatment. Cytotoxicity of PBL was examined using a non-radioactive cytotoxicity test. The figures show the lysis of two different target cells by PBLs on day 0, 22, 25 and 43. Target cells include K562, a CML cell line (**A**) and HLA-matched tumour cells (**B**). All experiments were performed in triplicates. Both figures represent data from ten experiments

1995, Schmidt-Wolf et al, 1997), may be suitable to remove residual tumour cells resistant to chemotherapy. Large numbers of these cells are necessary for effective immunotherapy. However, in some patients it is difficult to obtain sufficient effector cell numbers since these cells grow poorly in vivo.

Clinical trials on the use of gene-transfected lymphocytes were hampered by a poor efficiency of gene transfer in lymphocytes and a down-regulation of cytokine expression by lymphocytes (Hwu et al, 1993). The reason for this gene transfer resistance is not completely understood. Possibly, apoptosis plays a role in this gene transfer resistance of lymphocytes (Ebert et al, 1997). Retrovirus-mediated gene transfer is currently the method of choice for transfection of human T lymphocytes. Viral vectors, however, require dividing target cells (except for DNA-viruses such as adenoviruses) and may raise safety questions in human gene therapy (Schmidt-Wolf et al, 1996). CD3 receptor-mediated gene transfer is an efficient method for the transfection of CIK cells and cell mortality is relatively low, compared to other non-viral gene transfer methods (Buschle et al, 1995). However, it remains difficult to transfect large amounts of cells with this method. Electroporation is a fast and cost-effective method for transfection of large amounts of cells, but transfection efficiency is slightly lower compared to CD3 receptor-mediated gene transfer.

Here, we report on the results of the first trial using autologous IL-2 modified CIK immunological effector cells for the treatment of ten patients with metastatic disease. This pilot study demonstrates the feasibility and the low amount of toxicity of such an approach. Three patients developed fever, one patient developed anaemia. Two of the patients with fever received high amounts of non-vital cells. This may explain the fever in these patients that was self-limiting. After removing most of the non-vital cells with a second Ficoll gradient centrifugation, no fever was seen in the remaining patients.

There was a statistically significant increase in TGF-β in the serum of patients treated with CIK cells. Serum levels were not different in patients receiving CIK cells secreting high doses of IL-2 as compared to cells secreting low amounts of IL-2. TGF-β has been shown to inhibit the growth of renal cell lines (Ramp et al, 1997) and other human tumours (Markowitz et al, 1996). TGF-β promotes the growth and differentiation of dendritic cells (Riedl et al, 1997). On the other hand, aberrant expression of TGF-β has been proposed as a growth factor for malignant diseases (Wright et al, 1996). Therefore, the importance of the TGF-β increase is still unclear.

Interestingly, increased amounts of IFN-γ were measured in the serum of patients after treatment. There was no correlation between IL-2 levels of the cells and serum levels of IFN-γ. IFN-γ seems to be important for elevation of expression of MHC class II molecules improving the sensitivity of tumour cells against immunological effector cells. Similarly, GM-CSF has been described as being important for elevation of expression of MHC class II molecules. Interestingly, there was also a significant increase in GM-CSF in the serum of patients during therapy. A correlation existed in patient 10 between the serum cytokine level concerning IFN-γ and GM-CSF and clinical response. In patient 7 an increase of CD3+CD56+ lymphocytes was observed together with an increase of IFN-γ and GM-CSF in the serum.

There was a statistically significant increase in CD3+ T lymphocytes in the blood of patients during therapy. In accordance, there was an increase in cytotoxic activity in PBL derived from the patients that was partially significant. In three patients there was an increase in the response against recall antigens (Multitest Merieux$^{R}$) as compared to prior to vaccination. This may reflect an increase in efficiency of the immune system.

Clinical outcome in three patients showing no change of disease and one patient with complete response appears promising in patients with progressive metastatic disease resistant to chemotherapy. The patient with complete response only received CIK cells with a low amount of IL-2, but still responded clinically. In vivo, CIK cells have been shown to be effective in eradicating established tumours in mice (Lu et al, 1994). These data show that transfection is not absolutely necessary for effectiveness of CIK cells. However, in vitro data support the concept that CIK cells transfected with cytokine genes possess an augmented cytotoxic activity as compared to untransfected cells (Finke et al, 1998).

To our knowledge, this is the first report of the clinical use of CIK cells and of IL-2-transfected CIK cells. Further studies are necessary to evaluate the efficacy of such a treatment in particular in patients with limited disease.

*© 1999 Cancer Research Campaign*

### ACKNOWLEDGEMENTS

This work was supported by a grant from the Bundesministerium für Bildung, Wissenschaft, Forschung und Technologie, Bonn, Germany (01 KV 9542). The responsibility for the content is solely restricted to the authors. We thank Fa. Chiron, Ratingen, Germany for their kind gift of IL-2. We are grateful to Dr R Negrin, Stanford University for reviewing our manuscript.

### REFERENCES

Buschle M, Cotten M, Kirlappos H, Mechtler K, Schaffner G, Zauner W, Birnstiel ML and Wagner E (1995) Receptor-mediated gene transfer into human T lymphocytes via binding of DNA/CD3 antibody particles to the CD3 T cell receptor complex. *Human Gene Ther* **6**: 753–761

Csipai M, Lefterova P, Niemitz S, Johnston V, Scheffold C, Huhn D and Schmidt-Wolf IGH (1996) Effects of interleukin-7 on proliferation of tumour cells. *Cancer Res Ther Control* **5**: 11–16

Ebert O, Finke S, Salahi A, Herrmann M, Trojaneck B, Lefterova P, Wagner E, Kircheis R, Neubauer A, Huhn D, Schriever F, Wittig B and Schmidt-Wolf IGH (1997) Lymphocyte apoptosis: induction by gene transfer techniques. *Gene Ther* **4**: 296–302

Finke S, Trojaneck B, Lefterova P, Csipai M, Wagner E, Kircheis R, Neubauer A, Huhn D, Wittig B and Schmidt-Wolf IGH (1998) Increase of proliferation rate and enhancement of antitumour cytotoxicity of expanded human CD3+CD56+ immunologic effector cells by receptor-mediated transfection with the interleukin-7 gene. *Gene Ther* **5**: 31–39

Grimm EA, Mazumder A, Zhang HZ and Rosenberg SA (1982) Lymphokine activated killer cell phenomenon: lysis of natural killer resistant fresh solid tumour cells by IL-2 activated autologous human peripheral blood lymphocytes. *J Exp Med* **155**: 1823–1841.

Hickman CJ, Crim JA, Mostowski HS and Siegel JP (1990) Regulation of human cytotoxic T-lymphocyte development by IL-7. *J Immunol* **145**: 2415–2420

Hwu P, Yanelli J, Kriegler M, Anderson WF, Perez C, Chiang Y, Schwarz S, Cowherd R, Delgado C, Mule J and Rosenberg SA (1993) Functional and molecular characterization of tumour-infiltrating lymphocytes transduced with tumour necrosis factor alpha cDNA for the gene therapy of cancer in humans. *J Immunol* **150**: 4104–4115

Lotze MT, Grimm EA, Mazumder A, Strausser JL and Rosenberg SA (1981) Lysis of fresh and cultured autologous tumour by human lymphocytes cultured in T cell growth factor. *Cancer Res* **41**: 4420–4425

Lu PH and Negrin RS (1994) A novel population of expanded human CD3+ CD56+ cells derived from T cells with potent in vivo antitumour activity in SCID mice. *J Immunol* **153**: 1687–1696

Lynch DH and Miller RE (1990) Induction of murine lymphokine-activated killer cells by recombinant IL-7. *J Immunol* **145**: 1983–1990

Margolin KA, Negrin RS, Wong KK, Chatterjee S, Wright C, Forman SJ (1997) Cellular immunotherapy and autologous transplantation for hematologic malignancy. *Immunol Rev* **157**: 231–240

Markowitz SD and Roberts AB (1996) Tumour suppressor activity of the TGF-beta pathway in human cancers. *Cytokine Growth Factor Rev* **7**: 93–102

Mehta BA, Schmidt-Wolf IGH, Weissman IL and Negrin RS (1995) Two pathways of exocytosis of cytoplasmic granule contents and target cell killing by cytokine-induced CD3+ CD56+ killer cells. *Blood* **86**: 3493–3499

Ramp U, Jaquet K, Reinecke P, Nitsch T, Gabbert HE and Gerharz CD (1997) Aquisition of TGF-beta 1 resistance: an important progression factor in human renal cell carcinoma. *Lab Invest* **76**: 739–749

Riedl E, Strobl H, Majdic O and Knapp W (1997) TGF-beta 1 promotes in vitro generation of dendritic cells by protecting progenitor cells from apoptosis. *J Immunol* **158**: 1591–1597

Rosenberg SA, Spiess P and Lafreniere R (1986) A new approach to the adoptive immunotherapy of cancer with tumor infiltrating lymphocytes. *Science* **233**: 1318–1321

Rosenberg SA, Aebersold P and Cornetta K (1990) Gene transfer into humans: immunotherapy of patients with advanced melanoma, using tumor infiltrating lymphocytes modified by retroviral gene transduction. *N Engl J Med* **323**: 570–578

Schmidt-Wolf GD and Schmidt-Wolf IGH (1996) Cancer and gene therapy. *Ann Hematol* **73**: 207–218

Schmidt-Wolf IGH, Negrin RS, Kiem HP, Blume KG and Weissmann IL (1991) Use of a SCID mouse/human lymphoma model to evaluate cytokine-induced killer cells with potent antitumor cell activity. *J Exp Med* **174**: 139–149

Schmidt-Wolf IGH, Lefterova P, Johnston V, Huhn D, Blume KG and Weissmann IL (1994a) Propagation of large numbers of T cells with natural killer cell markers. *Br J Haematol* **87**: 453–458

Schmidt-Wolf IGH, Neubauer A, Finke S, Csipai M, Wittig B and Huhn D (1994b) Interleukin-7 gene therapy for patients with metastatic colon cancer, renal cell cancer, malignant melanoma or lymphoma. *Human Gene Ther* **5**(9): 1161–1168

Schmidt-Wolf GD, Negrin RS and Schmidt-Wolf IGH (1997) Activated T cells and cytokine-induced CD3+CD56+ killer cells. *Ann Hematol* **74**: 51–56

Welch PA, Namen AE, Goodwin RG, Armitage R and Cooper MD (1989) Human IL-7: a novel T-cell growth factor. *J Immunol* **143**: 3562–3567

World Health Organization (1979) *WHO Handbook for Reporting Results of Cancer Treatment*. WHO: Geneva

Wright JA and Huang A (1996) Growth factors in mechanisms of malignancy: roles for TGF-beta and FGF. *Histol Histopathol* **11**: 521–536

Zoll B, Lefterova P, Finke S, Trojaneck B, Ebert O, Micka B, Roigk K, Fehlinger M, Schmidt-Wolf GD, Huhn D and Schmidt-Wolf IGH (1998) Generation of cytokine-induced killer (CIK) cells using exogenous interleukin-2 (IL-2), -7 (IL-7) or -12 (IL-12). *Cancer Immunol Immunother* **47**: 221–226

*© 1999 Cancer Research Campaign*
